# Vaccination and Small Pox

**Published:** 1851-11

**Authors:** Andrew Nolen

**Affiliations:** Surgeon to Wicklow County Infirmary


*Vaccination and Small Pox.* By Andrew Nolen, Surgeon to Wicklow County Infirmary.—The interesting discussion on small pox at the Medico-Chirurgical Society, and the doubts there expressed of the security afforded by vaccination, induce me to submit the following cases for the consideration of the profession, as any facts, however isolated, which would tend to make vaccination more secure, are, in my opinion, of importance.

A young lady, about twenty, the second daughter of a gentleman residing in this neighborhood, after a country excursion was seized with rigors and other febrile symptoms, which eventuated in a smart attack of small pox: the pustules were numerous but distinct, on the face, chest, &c., and much fear was entertained lest she should be marked. She occupied a large airy apartment in a large mansion, and her mother and I relying on the security of very careful vaccination of the other young ladies, did not altogether prevent the ingress of her sisters, three of them were attacked, and two, the eldest and youngest escaped, though equally exposed; on inquiry of the ladies' mother, (a most intelligent woman,) she informed me that she recollected distinctly, that her eldest and youngest daughters had been tested by Bryce's method—i. e., re-vaccinated on the eighth day.

I mentioned this case lately to another lady, the mother of a large family, and she told me that her daughters had had small pox after vaccination, and that one who had been re-vaccinated on the eighth day, according to Bryce's plan, had escaped.—*The Lancet.*

